# Insulinoma with suspected mutant somatostatin receptor expression according to histological examination

**DOI:** 10.1002/ccr3.9390

**Published:** 2024-11-05

**Authors:** Shunichiro Onish, Aki Takada‐Watanabe, Robert Y. Osamura, Takayuki Shiomi, Hiroyuki Kusano, Yoshiro Maezawa, Hiroyuki Murai, Makoto Miyabayashi, Sakutaro Koike, Tomohiko Yoshida, Minoru Takemoto

**Affiliations:** ^1^ Department of Diabetes, Metabolism, and Endocrinology International University of Health and Welfare, Narita Hospital Chiba Japan; ^2^ Department of Endocrinology, Hematology and Gerontology Chiba University Graduate School of Medicine Chiba Japan; ^3^ Department of Diagnostic Pathology Nippon Koukan Hospital Kawasaki City Japan; ^4^ Department of Anatomic Pathology International University of Health and Welfare, Narita Hospital Chiba Japan; ^5^ Department of Neurology International University of Health and Welfare, Narita Hospital Chiba Japan; ^6^ School of Medicine International University of Health and Welfare Chiba Japan

**Keywords:** hypoglycemia, insulinoma, octreotide, somatostatin receptor

## Abstract

**Key Clinical Message:**

This case highlights the possibility of an insulinoma expressing an aberrant form of SSTRs resulting in a discrepancy between the preoperative octreotide assessment and postoperative SSTR expression.

**Abstract:**

Insulinoma is a pancreatic disease that causes hyperinsulinemic hypoglycemia. The first‐line treatment is surgery; however, somatostatin derivatives are administered in cases where surgery is not a viable option and to prevent preoperative hypoglycemia. Here, we report a case in which preoperative examination indicated a potential tumor with low somatostatin receptor 2 (SSTR2) and SSTR5 expression, whereas postoperative pathological examination indicated strong SSTR expression. We report the case of a 69‐year‐old Japanese female who experienced hypoglycemia‐like symptoms for a decade such as sweating, fatigue, hunger, and confusion with an increase in episode frequency per year. Dynamic computed tomography revealed a 13‐mm diameter nodule and aberrant blood flow in the pancreatic tail. Subsequently, the patient was diagnosed with pancreatic insulinoma. A preoperative octreotide test did not relieve hypoglycemia, and no uptake of ^111^indium‐pentetreotide was observed, suggesting an insulinoma with low SSTR expression. However, postoperative histological studies suggested that the intracellular domain of SSTRs were highly expressed, while the extracellular domain may be mutated. We present a rare case of insulinoma expressing an aberrant form of SSTRs resulting in a discrepancy between the preoperative octreotide assessment and postoperative SSTR expression.

## 1 INTRODUCTION

Insulinoma is a pancreatic disease that causes hyperinsulinemic hypoglycemia. Although the first‐line treatment is surgery, somatostatin derivatives are administered in cases wherein surgical options are not ideal and preoperative hypoglycemia needs to be managed.^1^ Preoperative octreotide testing can be useful for predicting somatostatin receptor (SSTR) expression in tumors.^2^ Herein, we report a case where preoperative examination indicated a suspected tumor with low SSTR2 and SSTR5 expression, whereas postoperative pathological examination revealed strong SSTR expression.

### 1.1 Case history/examination

A 69‐year‐old Japanese female had been experiencing hypoglycemia‐like symptoms such as sweating, fatigue, hunger and confusion for a decade, with a yearly increase in the frequency of hypoglycemic episodes. Patient had a history of jaw arthritis. The patient was referred for a detailed examination in our hospital. Findings on admission included a body height of 148.5 cm, weight of 44.9 kg, body mass index of 20.4 kg/m^2^, body temperature of 36.3°C, blood pressure of 174/91 mmHg, and a pulse rate of 70 beats per minute. Physical examination revealed the following: Mental status was clear; the bulbar conjunctiva was not pale and not icteric; there was no goiter, the skin was moist, and the skin turgor was not reduced. There was no abnormality on lung and heart auscultation; the abdomen was soft and flat, and there was no lower leg edema. Neurological examination revealed that the tendon reflexes were normal, and no pathological reflex was observed. Light and radial reflection was normal.

## 2 METHODS

During hypoglycemia, the blood glucose, insulin, and C‐peptide levels were 38 mg/dL (normal range: 70 ~ 109 mg/dL), 19.6 μU/mL (normal range: 5 ~ 15 μU/mL), and 3.54 ng/mL (normal range: 1.5 ~ 3.5 ng/mL), respectively, indicating hyperinsulinemic hypoglycemia that was treated with glucose. The patient tested negative for the insulin autoantibodies and did not take any medications that could cause hypoglycemia.

Dynamic computed tomography (CT) revealed a 13‐mm‐diameter nodule with abundant blood flow in the tail of the pancreas (Figure S1). Consequently, the patient was diagnosed with an insulinoma of the pancreatic tail, and an octreotide test was performed to prevent intraoperative hypoglycemia. Before the octreotide test, fasting blood glucose, insulin, glucagon, and growth hormone (GH) levels were measured. Next, 50 μg of octreotide was administered via subcutaneous injection to monitor all the parameters every 30 min for 120 min (Table 1). Glucagon and GH levels were inhibited by 84.6% and 84.9%, respectively. However, insulin was only inhibited by 19.8%, and hypoglycemia was not relieved. Additionally, there was no visible uptake of ^111^indium‐pentetreotide (octreotide scan) in the pancreatic tumor area (Figure S2). Therefore, insulinoma with low SSTR expression was diagnosed preoperatively.

**TABLE 1:** The results of octreotide test.

Time after octreotide load (min)	0	30	60	90	120
Blood glucose (mg/dL)	38	31	31	31	28
Insulin (μg/mL)	24.3	12.1	15.1	14.1	19.5
Glucagon (pg/mL)	26.0	< 3.5	< 3.5	< 3.5	4.0
GH (ng/mL)	2.52	0.80	0.43	0.36	0.38

*Note*: Fasting blood glucose, insulin, glucagon, and growth hormone (GH) levels were measured early in the morning, and octreotide (50 μg) was administered through subcutaneous injection to monitor each parameter every 30 min for 120 min.

## 3 CONCLUSION AND RESULTS

**FIGURE 1:**
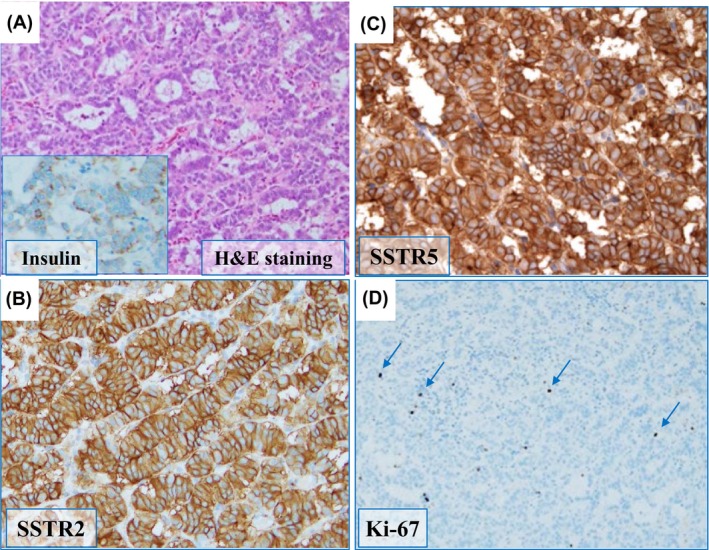
Histopathological images confirming insulinoma. Hematoxylin and eosin staining of the insulinoma and immunohistochemical image against anti‐insulin (inset) (A) Immunohistochemical staining for anti‐somatostatin receptor 2 (SSTR2), (B) anti‐SSTR5, (C) and anti‐ Ki‐67 antibodies, (D) in tumor cells, all of which are positive for insulin (A), SSTR2 (B), and SSTR5 (B) (cytoplasmic staining). The Ki‐67 labeling index of the tumor was 5% ((D) Arrows indicate Ki‐67 positivity), which indicates neuroendocrine tumor (NET) G2. The following antibodies were used for immunohistochemistry: Anti‐SSTR2 antibody and intracellular domain (ab134152, abcam), anti‐SSTR5 antibody and intracellular domain (ab109495, abcam), anti‐insulin antibody (DAKO), and anti‐Ki‐67 antibody (Roche).

A caudal pancreatectomy was performed and hypoglycemia resolved completely after tumor removal. Postoperative immunohistological examination showed a low Ki‐67 positivity rate of 5%; in contrast to preoperative expectations, SSTRs were highly enriched (score 3) in insulin‐positive cells (Figure 1).^3^ The subsequent pathological diagnosis was insulinoma, a well‐differentiated neuroendocrine tumor (NET) G2.

**FIGURE 2:**
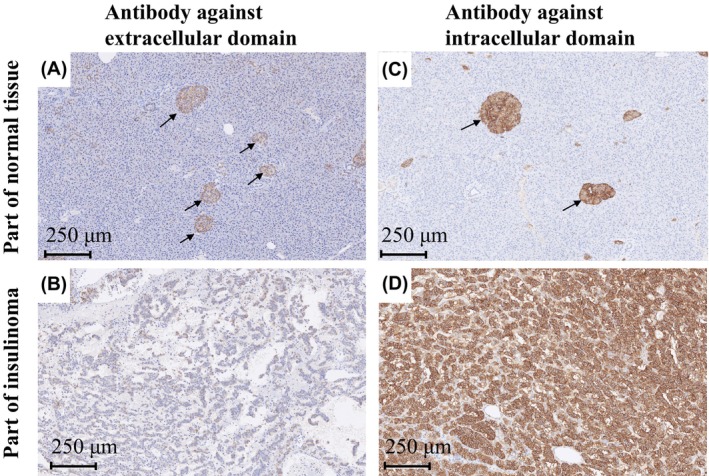
Histopathological image showing SSTR2 expression in the insulinoma. (A) Immunohistochemistry using anti‐SSTR2 antibodies targeted against the receptor's extracellular domain in the normal part of the pancreas. Arrows indicate cells in the islet of Langerhans. (B) Immunohistochemistry using anti‐SSTR2 antibodies against the receptor's extracellular domain in insulinoma. (C) Immunohistochemistry using anti‐SSTR2 antibodies against the receptor's intracellular domain in the normal part of the pancreas. Arrows indicate cells in the islet of Langerhans. (D) Immunohistochemistry using anti‐SSTR2 antibodies targeted against the receptor's intracellular domain in insulinoma. Histological studies indicate that the extracellular domain of SSTR2 expressed in insulinoma may be mutated compared to normal tissues. The following antibodies were used for immunohistochemistry: Anti‐somatostatin receptor 2 antibody; extracellular domain (ab140933, abcam), anti‐somatostatin receptor 2 antibody; intracellular domain (ab134152, abcam).

Discrepancies between preoperative and histological examinations of SSTRs warrant further analyses of SSTR expression. Examination of the antibody that recognizes the extracellular domains of SSTR2 and SSTR5 (Figure 2; Figure S3) showed recognition within the pancreatic islets; however, only faint signals from the insulinoma were detected. These results indicated no abnormalities in native SSTRs expressed by the pancreatic islets in this patient but showed insulinoma‐induced aberrant expression of extracellular SSTRs. Since aspartic acid 89 (89D) is crucial for ligand binding,^4^ we sequenced SSTR2 complementary DNA (cDNA) from insulinoma lesions that included 89D; however, no mutations were detected (Figure S4). These results indicate that the extracellular ligand‐binding domain of insulinoma was altered by post‐transcriptional modifications and not by simple gene mutations.

In conclusion, a case of insulinoma with negative results from an octreotide test and no octreotide scan uptake, but high SSTR expression in the tissue, was observed. The preoperative octreotide test correlates with SSTR expression in tissues; however, discrepancies can occur in certain cases, as in the present study.

## 4 DISCUSSION

Octreotide inhibits hormone production in endocrine organs and NETs via SSTR activity. SSTR2 expression in tumors correlates well with reactivity in the preoperative octreotide test.^2^ Octreotide scans have 90% sensitivity and 80% specificity for identifying pancreatic endocrine tumors using radioactive somatostatin analogs.^5^ Tumor expression of SSTRs is detectable if the tumor diameter is >1 cm. Negative octreotide scan results suggest reduced SSTR expression in the tumor.

Furthermore, in the octreotide test, glucagon and GH were sufficiently inhibited, whereas insulin was suppressed by only 19.8%. Consequently, the hormones mediated by SSTRs expressed in normal tissues can be suppressed by glucagon and GH. However, insulin was insufficiently suppressed. When combined with the octreotide scan results, a diagnosis of insulinoma with low SSTR expression was made. However, SSTRs were highly expressed on postoperative histopathological examination.

However, further histological studies showed that the intracellular domain of SSTR expressed in insulinoma was normal, but the extracellular domain was mutated.

In other words, because the extracellular domain of the insulinoma SSTR was mutated, it is presumed that the octreotide scan and octreotide tolerance test were negative in this patient.

There are reported cases of discrepancy between preoperative Octreotide scintigraphy and SST receptor expression. SSTR2 is expressed in most patients with a positive octreotide test. However, such expression was not observed in some cases, possibly because octreotide acts through SSTR5.^6^ Generally, SSTR2 expression is not observed in patients with a negative octreotide test. In our case, neither Octreoscan nor the octreotide tests were negative; however, although the extracellular domains were undetected, SSTR2 and SSTR5 intracellular domains were expressed.

SSTRs are G protein‐coupled receptors responsible for the anti‐proliferative activity of cells expressing the receptor when bound by the somatostatin‐14 agonist.^1^ Crucial amino acids, including D89, that are involved in stimulating intracellular responses upon ligand binding, have been characterized.^1^ Therefore, we expected that D89 may change because of genetic mutations however, the cDNA analysis did not support this hypothesis. The SSTRs expressed in the tumor may have undergone structural changes, likely due to post‐transcriptional modifications, reducing the affinity of octreotide, or the internalization of the extracellular domain of SSTRs.

Preoperative administration of Sandostatin causes a reduced expression of the SSTR extracellular domain which minimized the effect of Sandostatin (i.e., tachyphylaxis).^6^ This was not the explanation in our patient's case since we had not preoperatively prescribed Sandostatin and neither the octreotide test nor the octreotide scan was negative.

Future research should involve more in‐depth examination to understand why the extracellular domain of both SSTR2 and SSTR5 were changed.

Somatostatin analogs are widely used in treatment of acromegaly. However, there are cases in which Somatostatin is ineffective, and it is referred to as Somatostatin resistance. The mechanisms of Somatostatin resistance include impairment or heterogeneity of SSTR expression, mutation in SSTR genes and decreased sensitivity of SSTR proteins. A G protein gene mutation, particularly mutations in Gsα have been shown to be possible cause of SSTR decreased sensitivity.^7^ Therefore, G protein gene mutation might be happened in our case.

## AUTHOR CONTRIBUTIONS

**Shunichiro Onish:** Data curation. **Aki Takada‐Watanabe:** Data curation. **Robert Y. Osamura:** Methodology. **Takayuki Shiomi:** Methodology. **Hiroyuki Kusano:** Methodology. **Yoshiro Maezawa:** Data curation. **Hiroyuki Murai:** Data curation. **Makoto Miyabayashi:** Data curation. **Sakutaro Koike:** Data curation. **Tomohiko Yoshida:** Data curation. **Minoru Takemoto:** Writing – original draft.

## FUNDING INFORMATION

This work was supported in part by research grants from JSPS KAKENHI (Grant Numbers 23 K06889, 22 K07389, and 21 K07395).

## CONFLICT OF INTEREST STATEMENT

The authors declare that they have no competing interests.

## ETHICS STATEMENT

Ethics approval and consent to participate: This study was conducted in accordance with the ethical principles of the Declaration of Helsinki and approved by **I**nternational University of Health and Welfare's ethics committee.

## CONSENT

Written informed consent was obtained from the patient for the publication of this case report and any accompanying images. A copy of the written consent form is available for review by the editor‐in‐chief of the journal.

## Supporting information


Figure S1.
Figure S2.
Figure S3.
Figure S4.

## Data Availability

The data that support the findings of this study are available from the corresponding author upon reasonable request.
