# Supplementary material for: Insulinoma with suspected mutant somatostatin receptor expression according to histological examination
Source: Clin Case Rep. 2024 Nov 5;12(11):e9390. doi: 10.1002/ccr3.9390 (PMC11538038; doi:10.1002/ccr3.9390)
Supplement: Supplementary file 1 — Figure S1. Figure S2. Figure S3. Figure S4. [file CCR3-12-e9390-s001.pptx]

## Slide 1
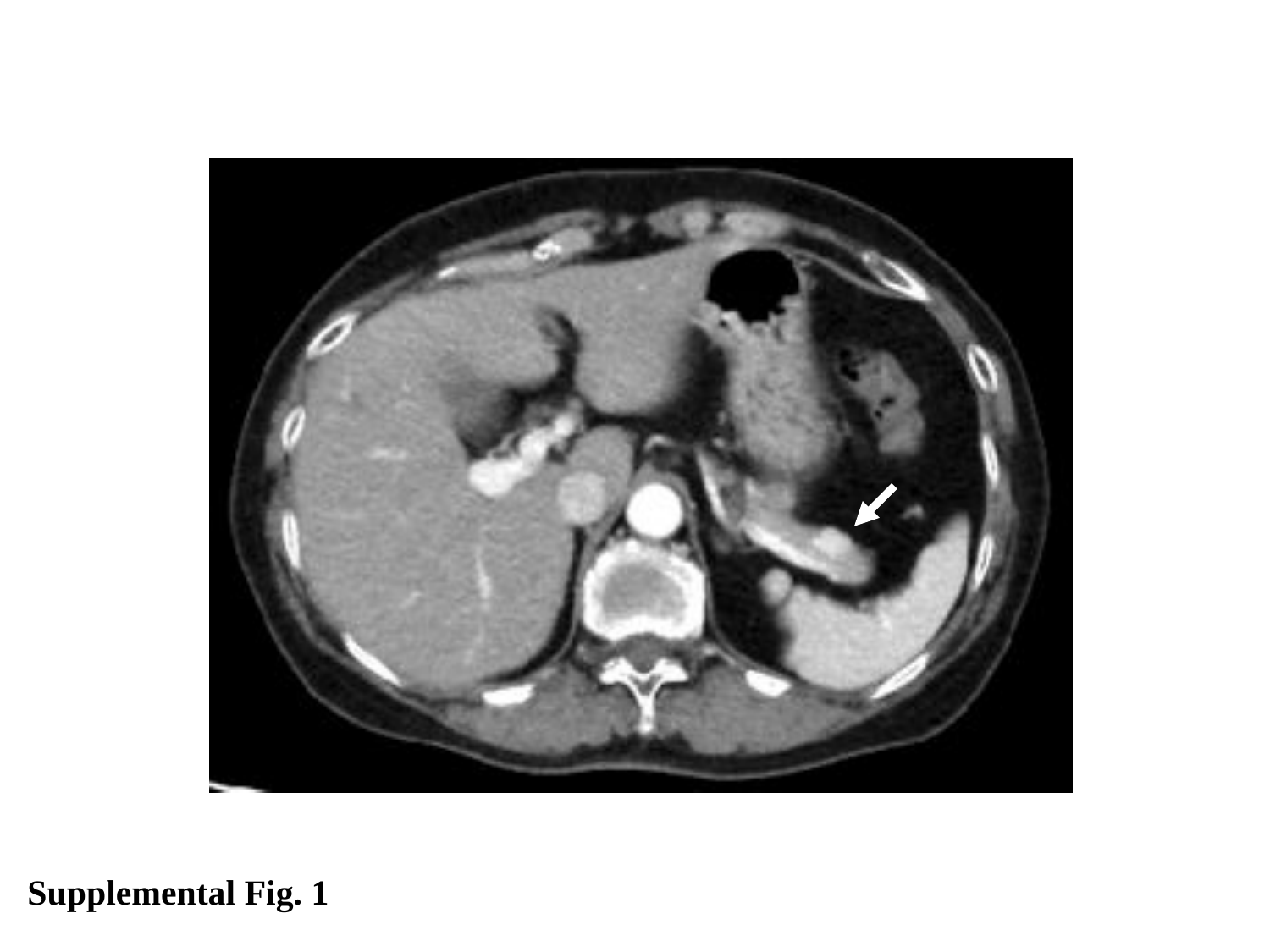

Supplemental Fig. 1

## Slide 2
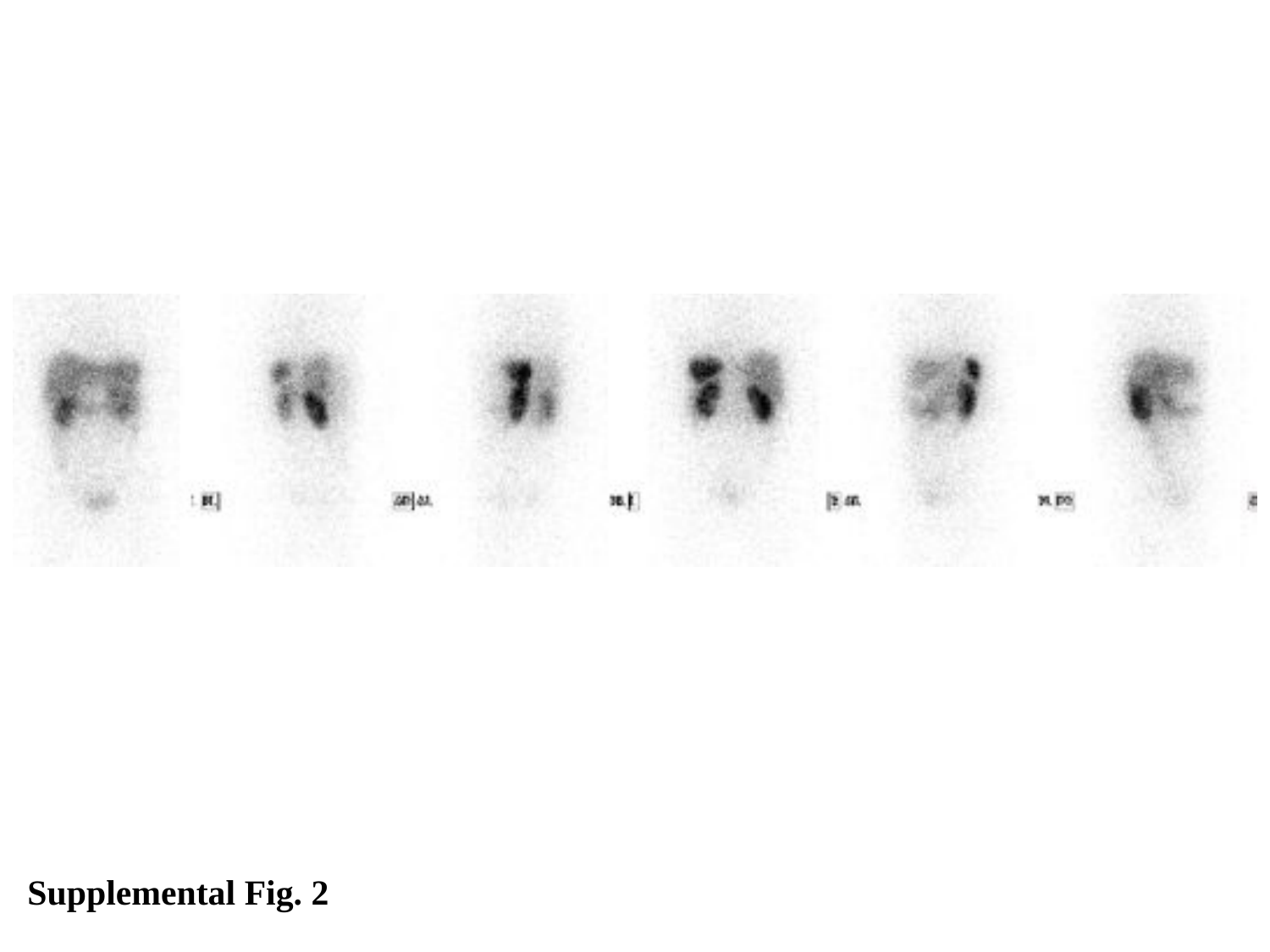

Supplemental Fig. 2

## Slide 3
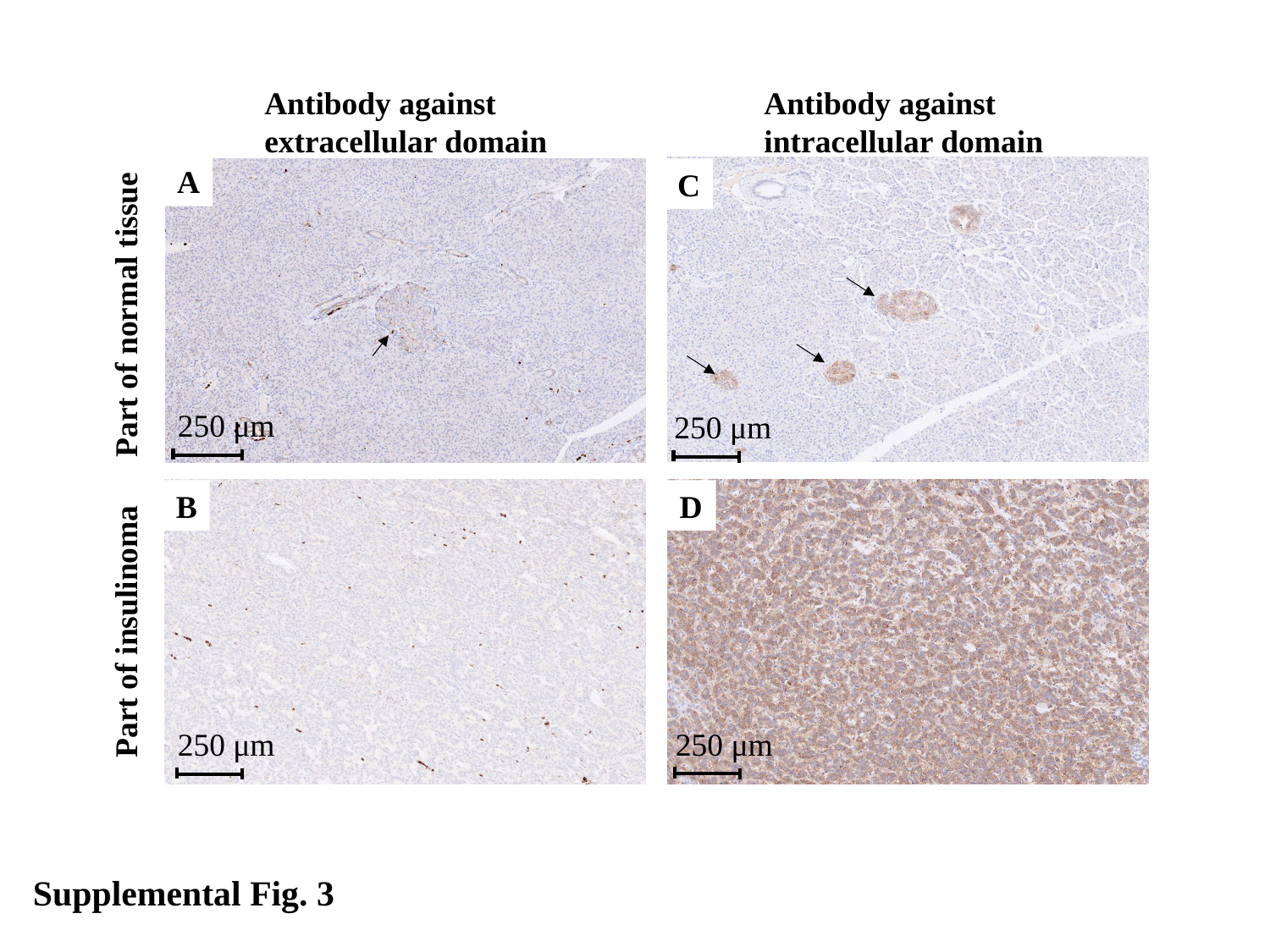

Antibody against
intracellular domain
Antibody against
extracellular domain
A
C
Part of normal tissue
250 μm
250 μm
B
D
Part of insulinoma
250 μm
250 μm
Supplemental Fig. 3

## Slide 4
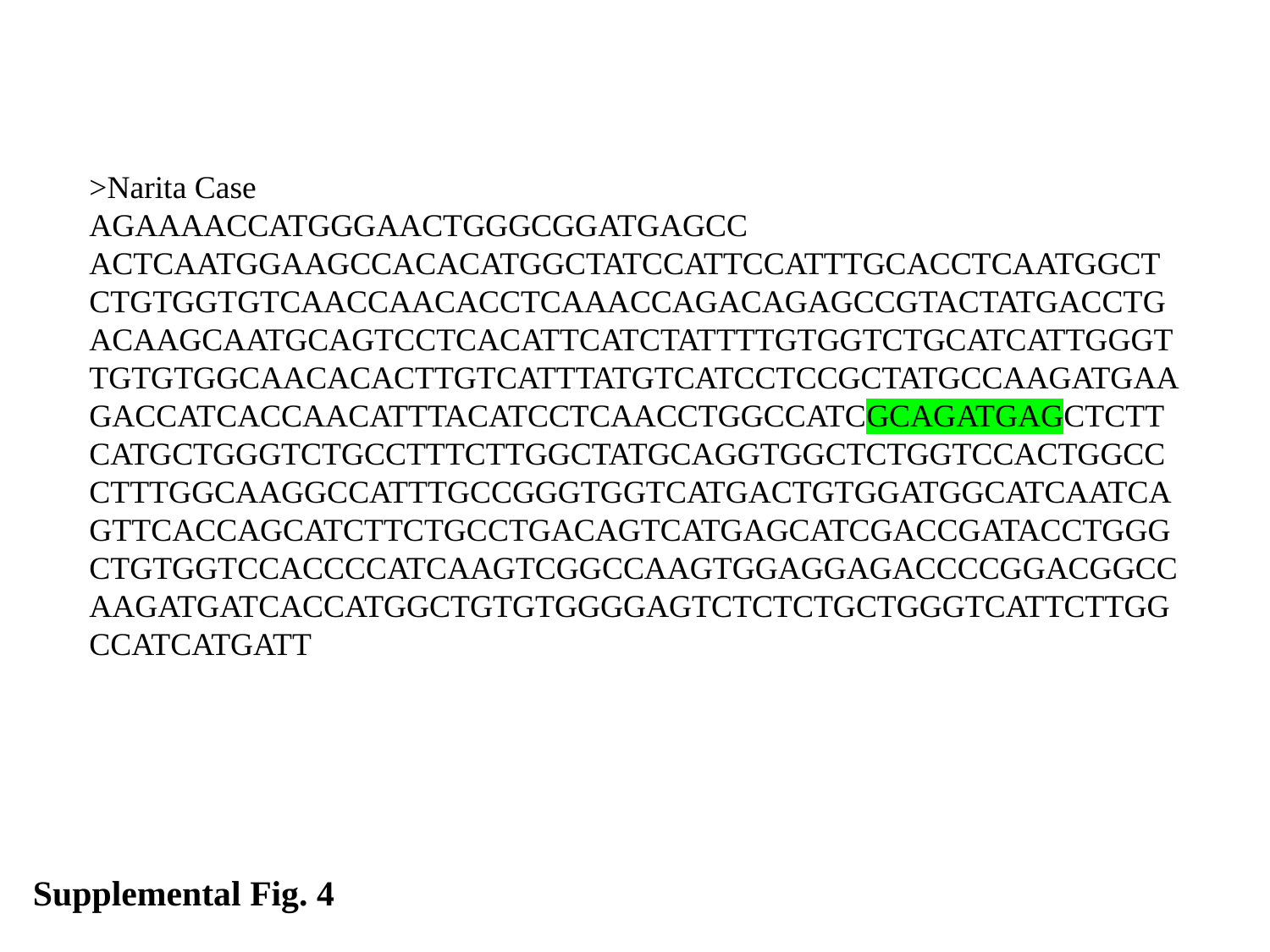

>Narita Case
AGAAAACCATGGGAACTGGGCGGATGAGCC ACTCAATGGAAGCCACACATGGCTATCCATTCCATTTGCACCTCAATGGCT
CTGTGGTGTCAACCAACACCTCAAACCAGACAGAGCCGTACTATGACCTGACAAGCAATGCAGTCCTCACATTCATCTATTTTGTGGTCTGCATCATTGGGTTGTGTGGCAACACACTTGTCATTTATGTCATCCTCCGCTATGCCAAGATGAAGACCATCACCAACATTTACATCCTCAACCTGGCCATCGCAGATGAGCTCTTCATGCTGGGTCTGCCTTTCTTGGCTATGCAGGTGGCTCTGGTCCACTGGCCCTTTGGCAAGGCCATTTGCCGGGTGGTCATGACTGTGGATGGCATCAATCAGTTCACCAGCATCTTCTGCCTGACAGTCATGAGCATCGACCGATACCTGGGCTGTGGTCCACCCCATCAAGTCGGCCAAGTGGAGGAGACCCCGGACGGCCAAGATGATCACCATGGCTGTGTGGGGAGTCTCTCTGCTGGGTCATTCTTGGCCATCATGATT
Supplemental Fig. 4
